# IT MAKES ME FEEL LIKE I’M NOT ALONE: GRANDPARENT CAREGIVERS’ DISCUSSIONS OF SOCIAL SUPPORT

**DOI:** 10.1093/geroni/igz038.1040

**Published:** 2019-11-08

**Authors:** Jessica Freeman, Jessica Elton

**Affiliations:** 1 University of Tennessee at Chattanooga, Chattanooga, Tennessee, United States; 2 Eastern Michigan University, Ypsilanti, Michigan, United States

## Abstract

This study investigated skipped-generation “grandfamily” caregivers’ sources of social support. Skipped-generation “grandfamilies” are defined as families in which grandparents co-reside with and take on parental responsibilities for grandchildren (Shakya, et al., 2012). Nearly 2.6 million U.S. grandparents are responsible for at least one grandchild (Generations United, 2017). How grandparent caregivers seek social support is an important topic of research because social support has the potential to influence health, personal relationships, and sense of self-worth (Burleson, 1990, 2003; Sarason & Sarason, 2009; Vangelisti, 2009). Thus, this exploratory study looked at if, why/why not, and how skipped-generation grandparents seek social support. The study also elaborated upon which types of social support grandparent caregivers described receiving. Interviews were conducted with grandparents (N = 21) who identified as primary caregiver for at least one grandchild. Two independent coders analyzed transcripts, applying Braun and Clarke’s (2006) approach to thematic analysis. Results revealed that several grandparent caregivers report a sense of isolation and do not seek out formalized support structures due to lack of time, sense of connection, or interest. On the other hand, others receive social support formally and informally, via a number of channels including support groups (online and in-person), trained professionals, and friends/family. Following Cutrona and Suhr’s (1992) social support categorization, the results indicate that grandparent caregivers most often seek support falling into categories ranging from informational/advice, emotional, tangible, and networking.

